# Transactions of the Illinois State Medical Society for 1852

**Published:** 1852-10

**Authors:** 


### EDITORIAL.

*Transactions of the Illinois State Medical Society for* 1852.

In a previous number we published a brief account of the proceedings of the Illinois State Medical Society, at its annual meeting held at Jacksonville on the 1st of June last. Since then we have received a copy of the published transactions, containing, in addition to the minutes of the meeting, the valedictory address of the retiring president, Dr. S. Thompson, a report from the Committee on Practical Medicine, and several interesting papers presented by members of the society.

The valedictory address of Dr. Thompson is what might have been expected from one who for many years has been devoted to the study, as well as practice, of his profession; well written and filled with high-toned sentiments and valuable suggestions. One of the principal objects in the address is to impress upon the members of the society, and others of the profession, the importance of availing ourselves of what is already known and established, and the dangers to be apprehended from a neglect of old truths, in our love for what may purport to be newly discovered facts and recently expressed views and opinions.

No worthy member of our profession can fail to appreciate and fully endorse the views expressed in the two following concluding paragraphs of the address :—

"The objects of our society are to gain knowledge, to draw the line between knowledge and ignorant pretence.—To make the acquirements and experience of all be for the benefit of each.—To supply the non-professional public not only with better advice, in their sufferings, but to mark the line by which they may be guided in selecting those to whom to entrust their health.

"We can only do this by our own eminence in skill and knowledge. A practiced eye can distinguish slight shades of difference in the size or color of objects; but to those who in such matters cannot possess this acumen we must be sure there are no approximating colors. We must be white as against black; high as contrasted with low; learned, in fact, not ignorant smatterers. Thus shall we mark the distinction between the physician and the quack, by an appeal to the sense of the community, and their own interest, which is above all legal distinctions, or legal protections, either for the people, or the profession."

The report of the Committee on Practical Medicine by its chairman, Dr. N. S. Davis, being, as it is, made up from materials derived exclusively from the views, and results of the observations and experience, published during the year by Physicians of Illinois; is particularly valuable and interesting to the practitioners of this and the neighboring States.

Much credit is due to this committee for showing, by the result of their efforts to embody in this report the facts and remarks which a *few* have recorded, what might be accomplished if *more* of our physicians would do themselves and the State justice, and be faithful to the interests of the profession, by publishing from time to time (in our *Journal* of course) the results of their observations and experience.

Much of the matter contained in the report has been taken in the form of abstracts and quotations from this *Journal*, and has been the means of eliciting from the committee facts and remarks, many of which are especially interesting when taken in connection with the published articles to which reference is made.

Under the head of "opium in large doses," is a somewhat extended notice of Dr. A. G. Henry's paper, on its use in dysentery and fever, at the conclusion of which, Dr. Davis adds the following as the result of his experience :—

"Having seen much of the typhus and typhoid fevers in New York, and the intermixture of these types with our common remittents in this city, and especially among the poorer classes admitted into the medical wards of the Illinois General Hospital, we are satisfied that nothing could be more injurious than the indiscriminate application of any '*plan*' of treatment to all the forms and varieties of fever, whether such *plan* consists in the exhibition of large doses of opium or anything else. That the exhibition of four or six grains of opium immediately after a free evacuation of the alimentary canal by calomel and ipecac will often throw the patient into a profuse sweat, and cut short an ordinary bilious or remittent fever, I have no doubt. That it will occasionally fail, however, and induce a most dangerous degree of stupidity or narcotism, I am equally certain. While in typhoid cases, complicated as they generally are with intestinal or bronchial irritation and ulceration, the *large* doses of opium will not *occasionally* but generally fail to produce any favorable impression whatever. And in one or two cases, coming under my own observation, they produced manifestly fatal results. Instead of regarding all the types of fever as amenable to one *plan* of treatment, we are confident that much of the skill and success of the practitioner will depend on the accuracy with which he discriminates the type, tendency, and complication of each individual case."

After quoting from an article translated from the French, for the November number of this *Journal*, on the use of the extract of beef's blood as a remedial agent, the chairman of the committee gives the following as the result of his experience :—

"Often meeting with cases of protracted and extreme anæmia in the wards of the Illinois General Hospital, the chairman of your committee prepared a quantity of the dried blood, and exhibited it in several strongly marked cases of anæmia arising from different causes. In most of the cases its effects were most salutary and gratifying. One of the most striking cases was a young man, brought into the Hospital for a supposed affection of the heart. His whole surface appeared bloodless; his tongue and lips pallid; his pulse small, quick, and easily compressed; he had giddiness, frequent palpitations, especially on making an attempt to sit up, or walk. The bowels were slightly costive, and appetite indifferent. Auscultation revealed the usual bellows murmur of extreme anæmia, but no symptoms of organic disease. He stated that some weeks previous he had suffered from a protracted course of periodical fever. He was ordered twenty grains of the extractum sanguinis, or dried blood, every six hours, and a simple but nutritious diet. Under this course he improved so rapidly that in little more than two weeks he left the Hospital, and resumed his work as a laborer."

"Cerebro-spinal arachnitis" is noticed in the report, in connection with remarks upon the articles published in our *Journal* upon this subject by Dr. James Smick, Dr. J. B. Nash, and Dr. J. S. Whitmire.

The following quotation contains the substance of the views expressed by the committee, with regard to the articles referred to, and the nature of the disease under consideration :—

"We think every reader of much experience in our periodical fevers will at once recognise those cases as simple *congestive intermittents*, in which the brain and spinal cord were specially involved. Founding his opinion on the observation of those cases, Dr. Whitmire was undoubtedly right in regarding them as destitute of inflammation, and to be successfully treated only by the most prompt administration of anti-periodics in full doses. His error, if any, consisted in applying to them the name of 'Cerebro-Meningitis,' from which they manifestly differ widely in many essential symptoms, as well as in their progress and final termination.

"*First.* The cases of Dr. Whitmire were 'strictly periodical,' the patients usually becoming quite comfortable between the paroxysms; while in true cerebro-spinal meningitis, if the accompanying fever remits, the symptoms of cerebral disease remain well marked until the case terminates in the beginning of convalescence or in death.

"*Second.* In the true meningeal disease, the effusion found on post mortem examination is not merely 'bloody serum,' as represented by Dr. Whitmire, but consists of more or less purulent matter, and often a thick layer of well formed pus over the whole surface of the pia-mater and arachnoid; the membranes themselves bearing all the marks of intense inflammation. Thus, Drs. Smith and Payne, in making a post mortem examination of one of their fatal cases, found, 'on opening the cranium, the external blood-vessels were highly congested; the arachnoid was inflamed in every part of it, and covered with a *thick layer of pus.*' Such appearances could not arise from mere 'congestion or paralysis of the cerebral vessels.' Hence we must agree with all those who have examined fatal cases, that the cerebro-spinal disease is not only inflammatory, but most rapidly and intensely so.

"The principal objections to Dr. Smick's views in reference to the malarious origin of the disease, are its occasional prevalence in districts of country and at seasons of the year generally exempt from the prevalence of malaria, and its entire absence in other districts and seasons when all the circumstances supposed by Dr. Smick to favor its development are present in a high degree. From the facts detailed by Dr. Smick it is evident that the localities in which he has observed the disease were highly malarious, and from the symptoms detailed it is equally evident that whether *malaria* was the essential cause or not, this influence constituted an important element in its character: and hence that the early resort to quinine and powerful revulsives constituted an important part of the treatment; just as they do in pneumonia when it occurs in localities highly favorable to the prevalence of periodical fevers. In all such instances great caution is required to prevent overlooking the primary or essential causes and pathological conditions in view of those that are accidental or merely modifying."

Embodied in the report are two short articles upon "the progress of epidemics," one by Dr. H. Shoemaker, of Monroe Co., the other from Dr. J. T. Stewart, of Peoria.

The following quotation from the paper of Dr. Shoemaker contains the substance of his remarks upon epidemic dysentery, as it prevailed in Southern Illinois during the last year:—

"As regards epidemics in Southern Illinois during the last twelve months, so far as has come to my knowledge, two only have appeared; these were of quite a different character; the first, dysentery of a very grave type, usually accompanied by considerable hæmorrhage, and very obstinate to treatment. So great was the flux of blood in many that at an early period I had recourse to the mineral tonics; among which the oxide of silver, so highly commended by Sir James Eyre, and others. I must confess myself disappointed in the result. The nitrate of silver in solution, in starch enema, was found of more marked benefit. These remedies were of course used with, or subsequent to, the exhibition of mercurial alteratives. The most certain treatment was the old one of minute doses of calomel or hydrarg. cum creta, and a plentiful supply of solid opium, anodyne fomentations to the abdomen, starch and nitrate of silver injections. When toleration was established, free doses of Dover's powder were of marked benefit; and where a disposition to paroxysmal returns was manifested quinine was had recourse to; where there was a proclivity to anæmia, (a thing very common after fluxes of blood,) the citrate of quinine and iron combined with small doses of pulv. Rhei, say five grains of the latter and three of the former, given every four or five hours, had an excellent effect."

Our readers may learn what constitutes Dr. Stewart's treatment of the above-named disease, from the following quotation from his paper:—

"In the dysentery that followed the cholera nothing peculiar was noticed except a tendency, in the month of October, to assume a periodical character, in which cases it yielded to sulph. quinine. I found a combination of sulph. morphia, gum camphor and calomel act very promptly in arresting this disease in many cases; but the treatment I usually found most effectual was a powder every four hours of calomel gr. j. a. ij., gum camphor gr. ij. a. iij., sulph. morphia gr. qr., pulv. ipecac gr. ss., alternating with a table-spoonful of the following mixture: ol. Ricini scru. j., pulv. G. acaciæ dr. ij., sacch. alb. dr. iij., aq. menth. scru. iv. Some cases which had resisted other treatment for a considerable time yielded readily to the simple oleaginous mixture given in table-spoonful doses every four hours."

Dr. Thos. Hull, one of the committee on practical medicine, made a report in addition to the one made by the chairman, in which he gives his views and the result of his experience in the treatment of a severe form of epidemic dysentery, as it prevailed in the Northern part of the so-called Military district, as follows:

"My view of the matter is simply this, that the epidemic of last year was a miasmal disease, a modification merely of our autumnal fevers, or, in the language of Sydenham, 'the dysentery I speak of is the very fever itself, with this particularity, that it is turned inwards upon the intestines, and discharges itself that way.' He has also recorded an 'epidemic looseness' that precedes the dysentery, which was ushered in by 'chillness and shaking,' injured by rhubarb and astringents, successfully treated by bleeding and a cool regimen; and he goes on to say, 'this disease, though naturally gentle, frequently proved mortal.'

"The first impression of the exciting cause being made on the organic nerves, a diminution or loss of nervous energy and vital power is the consequence, and which in ordinary years would be followed by chill and fever, but owing to other causes, concurring and determining, is deflected from its usual course. The successive effects probably take place in the following order: congestion of the liver, (biliary and venous,) of the spleen, of the mucous membrane of the bowels, particularly of the descending colon, irritation, inflammation, suppuration and ulceration, gangrene and death.

"Admitting this view to be correct, the indications for sound treatment were plain, and were promptly and fearlessly carried into effect. The first patient in which it was adopted was an intelligent individual. I explained to him my views of the disease, and the way I wished to treat him. He at once consented. In this case mucous diarrhœa had existed about twenty-four hours, and for some five or six hours the discharges had increased in frequency, and were mixed with blood. The countenance anxious, skin sallow, tongue loaded, liver tender, spleen enlarged, and unusually sensitive; the ascending, transverse, and a small portion of the descending colon loaded, but no uneasiness felt, on pressure; the remaining portion extremely sensitive; and a constant harassing desire to micturate and empty the bowels. Thirty grains of calomel, three of ipecacuan., and a quarter of a grain of the muriate of morphine were given, followed, in three hours, by two ounces of castor oil, and thirty minims of laudanum. In a little over two hours after taking the oil he had a copious fæcal evacuation, with mitigation of all the distressing symptoms. This was quickly followed by others, but of a more bilious character. Nitrate and bi-nitrate of potash were dissolved in water as a drink. Every trace of the disease had vanished from the discharges in four hours from the operation of the medicine. Some tenderness remained in the descending colon, but much diminished: the spleen perceptibly lessened, and it and the liver less sensitive. Twenty grains of quinine, and a quarter grain of morphine was given at bed-time. The bowels were subsequently kept gently open for a few days with oil. Quinine in common doses was continued, and the disease never returned.

"This treatment was carried out in every succeeding instance, one only excepted, and the result is here given in a tabular form. The number of days includes the day on which the medicine was given, and succeeding days, until no mucous trace existed in the alvine discharges. I will here remark that considerable alarm existed in the public mind at this time; and I believe that every case was treated, if not seen, on the day when blood was first discovered in the stools. It will be noticed that in six cases the disease was crushed in twenty-four hours, or less, that is, on the day after the medicine was taken the discharges were free from blood and mucous.

| CASES. | DURATION. | |
|---|---|---|
| 6 | 1 day, | 6 |
| 19 | 2 | 38 |
| 12 | 3 | 36 |
| 1 | 4 | 4 |
| 1 | 5 | 5 |
| 1 | 6 | 6 |
| 40 | | 95 av'ge $2\frac{1}{3}$ days. |

"In these cases every symptom of dysentery was present. I have purposely omitted all where no blood was visible in the discharges, although I believe that if 'the discharges be frequent, mucous, and accompanied with gripings, the distemper may as justly be termed dysentery as if the blood was discharged with them.' That is, during the prevalence of the 'dysenteric constitution.'"

In addition to matter embraced in the reports of committees, we find in the published transactions under consideration three papers, two from Dr. E. S. Cooper and another from Dr. L. C. Lane, both of Peoria.

Dr. Cooper deserves much credit, and is entitled to the thanks of the profession for his two interesting papers, describing surgical instruments which he has invented, one for cauterizing the urethra, the other for treating "incomplete anchylosis of the knee joint." If, after being tested, the last named instrument should prove as useful in the hands of others as in those of the inventor, as appears from the history of several cases reported by him, it must come into general use, for the treatment of numerous cases previously considered as very intractable, if not incurable.

Dr. L. C. Lane in a short paper, embracing the history of a case to illustrate his views, contends that lacerated and contused should be changed into incised wounds, by cutting off the lacerated and contused portions. This, doubtless, is the proper treatment, yet we are not aware that there is anything original in the suggestion. All good surgeons, we believe, should make it a rule to remove parts so much injured as to endanger their vitality. H.

